# Understanding PrEP decision making among pregnant women in Lilongwe, Malawi: A mixed‐methods study

**DOI:** 10.1002/jia2.26007

**Published:** 2022-09-08

**Authors:** Lauren M. Hill, Carol E. Golin, Friday Saidi, Twambilile Phanga, Jennifer Tseka, Alinda Young, Lisa D. Pearce, Suzanne Maman, Benjamin H. Chi, Wilbroad Mutale

**Affiliations:** ^1^ Department of Health Behavior University of North Carolina at Chapel Hill Chapel Hill North Carolina USA; ^2^ Department of Medicine University of North Carolina at Chapel Hill Chapel Hill North Carolina USA; ^3^ UNC Project‐Malawi Lilongwe Malawi; ^4^ Department of Obstetrics and Gynecology School of Medicine University of North Carolina at Chapel Hill Chapel Hill North Carolina USA; ^5^ Department of Maternal and Child Health University of North Carolina at Chapel Hill Chapel Hill North Carolina USA; ^6^ Department of Sociology University of North Carolina at Chapel Hill Chapel Hill North Carolina USA; ^7^ Department of Health Policy University of Zambia School of Public Health Lusaka Zambia

**Keywords:** HIV, PrEP, decision‐making, pregnancy, women, Malawi

## Abstract

**Introduction:**

Pre‐exposure prophylaxis (PrEP) is a promising tool for HIV prevention during pregnancy. With increasing rollout in antenatal settings, counselling strategies to help pregnant women make appropriate decisions about PrEP use are needed. Understanding women's motivations and concerns for PrEP use—and how these inform their decision making and feelings about the decision to start PrEP—are critical to inform these strategies.

**Methods:**

We conducted a convergent mixed‐methods study from June 2020 to June 2021 in the context of a PrEP adherence support trial among HIV‐negative pregnant women in Lilongwe, Malawi. Two hundred women completed a survey reporting their motivations and concerns about PrEP use, and their feelings about the decision to start PrEP (Decisional Regret Scale). Thirty women completed in‐depth interviews to better understand the decision‐making process, including motivations and concerns weighed in women's decision to use PrEP. Analyses comprised descriptive and bivariate statistics, thematic qualitative analysis, and integration of quantitative and qualitative results.

**Results:**

Women initiating PrEP during pregnancy were highly motivated to obtain HIV protection for themselves and their unborn child, often due to perceived HIV risk connoted by a recent sexually transmitted infection and/or concerns about partner non‐monogamy. These motivations prevailed despite some concerns about safety and side effects, anticipated stigmatization, and concerns about adherence burden and pill attributes. Many women had informed their partner of their decision to use PrEP yet few felt their decision was contingent upon partner approval. Most women felt positively about the decision to start PrEP (mean decisional regret = 1.2 out of 5), but those with a greater number of concerns reported greater decisional regret (B = 0.036; *p* = 0.005). Furthermore, women who were specifically concerned about partner disclosure, who disliked pills or who had no perceived HIV risk reported greater decisional regret.

**Conclusions:**

Pregnant women were strongly motivated by the promise of HIV protection offered by PrEP and accepted it despite diverse concerns. A shared decision‐making approach that centres pregnant women and offers partner involvement may help identify and address initial concerns about PrEP use and support prevention‐effective use of PrEP during this important period.

## 1 INTRODUCTION

Pregnancy and postpartum are critical periods for preventing horizontal and vertical transmission of HIV in sub‐Saharan Africa. Women are at elevated HIV risk during this time due to increased biological and behavioural risk factors [1, 2, 3, 4, 5]. An estimated one‐third to one‐half of mother‐to‐child transmission (MTCT) of HIV is attributable to acute maternal infection [6, 7, 8]. Pre‐exposure prophylaxis (PrEP) is a proven yet substantially underutilized tool to address maternal HIV infections and eliminate MTCT. Daily oral PrEP with tenofovir‐emtricitabine is highly efficacious in preventing HIV infection with high adherence [9, 10, 11], and safe to use during pregnancy and breastfeeding [12]. World Health Organization guidelines recommend that PrEP be offered in standard PMTCT practice [13].

Despite the promise of PrEP, significant implementation challenges exist [14]. Evidence suggests that PrEP may be highly acceptable among PrEP‐naïve women, yet attrition and non‐adherence are significant challenges for women using PrEP [15, 16, 17, 18, 19]. Women in PrEP trials have had consistently poor adherence despite adherence support [20]. Promoting user fit rather than adherence support alone may be critical to achieve prevention‐effective PrEP use among pregnant and breastfeeding women.

To promote user fit (concordance of user values with the features of the chosen option) for preference‐sensitive medical decisions like the choice to use PrEP, patient‐centred counselling approaches like shared decision‐making (SDM)—a process by which patients and clinicians work together to make healthcare decisions—are recommended [21]. Few SDM or other patient‐centred approaches for PrEP decision counselling exist; those developed to date have focused on male and non‐pregnant US‐based populations [22, 23]. A better understanding of women's values and preferences for PrEP decision‐making in the context of pregnancy is needed to develop patient‐centred counselling approaches.

To address this gap, we conducted a mixed‐methods study among pregnant women in Lilongwe, Malawi who had recently decided to start PrEP. We sought to understand women's motivations and concerns for PrEP use, and how these affected their feelings about the decision. We further assessed how they involved partners and family members in their decision. The results of this study will inform SDM strategies to support pregnant women's decision‐making about PrEP use.

## 2 METHODS

### 2.1 Study context

Data were collected from June 2020 to June 2021 in a pilot trial of an adherence support programme for women using antiretroviral drugs (ARVs) for either HIV treatment or prevention during pregnancy and breastfeeding. The intervention and pilot study are described elsewhere [24, 25]. The sub‐study presented here was nested within the Tonse Pamodzi 2 (TP2) PrEP trial that enrolled pregnant women at risk of HIV acquisition interested in initiating daily oral PrEP (*n* = 200). HIV‐negative pregnant women were recruited from Bwaila District Hospital in Lilongwe, Malawi if they met any of these PrEP indications: HIV‐positive partner or unknown partner HIV status, multiple sex partners, sexually transmitted infection (STI) diagnosis, use of post‐exposure prophylaxis, use of shared injection equipment or an unspecified HIV risk concern (for full eligibility criteria, see [24]).

All women were counselled about their HIV risk and how PrEP could reduce their risk. During informed consent, they received information on the function of PrEP, the importance of adherence, side effects and safety. Women were prescribed PrEP at enrolment and given further education about how PrEP works, dosing and efficacy, duration of use and adherence strategies.

### 2.2 Quantitative data collection

HIV‐negative pregnant women in the TP2 PrEP trial (*n* = 200) completed an interviewer‐administered survey at enrolment. We collected information about PrEP features and individual and interpersonal factors that had motivated or concerned them when deciding to use PrEP; women were asked if they had considered each of seven common motivators and nine common concerns for PrEP use when they decided to use PrEP (motivations and concerns identified through literature review; yes/no response). We assessed their feelings about the decision to use PrEP through the five‐item Decisional Regret Scale, measuring “distress or remorse over a decision” [26]. Participants rated each item on a five‐point Likert Scale (1‐Strongly Agree to 5‐Strongly Disagree), with greater mean scores reflecting greater regret about the decision to use PrEP. The scale showed acceptable internal consistency in the study sample (Cronbach's alpha = 0.65). Decisional regret was assessed after women made the decision to use PrEP (at screening which typically occurred 1–3 weeks before enrolment), but before starting PrEP. This timing was chosen because we anticipated the differential effects of intervention counselling on perceptions of decisional regret. Decisional regret responses in this study reflect women's feelings about the decision to use PrEP after having time to consider their initial decision, but before initiating PrEP.

### 2.3 Qualitative recruitment and data collection

We conducted 30 in‐depth interviews (IDIs) with women participating in the TP2 PrEP trial. Women were purposively recruited to ensure variation in partner HIV status. Women completed the IDI an average of 102 days after enrolment (range: 59–239 days) and interviews lasted 25–40 minutes. IDIs were conducted in a private room by a research assistant (RA) fluent in Chichewa and English using a semi‐structured interview guide (different RAs than survey interviewers). Interview topics included: PrEP decision‐making process; values that informed decision‐making; reasons for accepting PrEP; the perceived difficulty of the decision; and the involvement of partners and others in this decision. Each interview was digitally recorded, transcribed and translated into English.

### 2.4 Ethical considerations

Study procedures were approved by the Malawi National Health Science Research Committee and the University of North Carolina at Chapel Hill Institutional Review Board. The TP2 trial was registered on www.clinicaltrials.gov (NCT04330989). All participants provided written informed consent prior to study procedures. For illiterate participants, a literate impartial witness was present during the consent process.

### 2.5 Analysis

Quantitative analyses included: generating descriptive statistics, including means (SD) and frequencies (percentages); assessing bivariate associations of each motivation or concern with decisional regret using Mann–Whitney tests; testing associations between the total number of motivations and concerns and decisional regret with unadjusted linear regression.

The thematic qualitative analysis consisted of: (1) reading transcripts and noting emerging themes; (2) creating a codebook, including structural codes (corresponding to interview topics) and interpretive codes (corresponding to emerging ideas); (3) coding with 20% of transcripts double‐coded by independent coders who reconciled discrepancies prior to further coding (coding by interviewers and study coordinator); (4) summarizing responses pertaining to each topic/code in matrices to facilitate by‐topic summary and comparisons across participants; and (5) reviewing summaries to identify principal themes and observe the variation within each theme.

To better understand the salience of women's motivations and concerns in their decision to use PrEP, quantitative and qualitative results were integrated following a convergent mixed‐methods approach [27]. Results from each source were compared in a joint display matrix to find points of convergence, divergence or added insight. We observed which values were most important for women's decision‐making by noting quantitative prevalence, association with decisional regret and qualitative salience for PrEP decision‐making.

## 3 RESULTS

### 3.1 Sample description

Two hundred HIV‐negative pregnant women completed the survey. Participants were 25 years old on average (Table 1). Most had an HIV‐negative primary partner (75%), 20% did not know their partner's HIV status and 5% reported an HIV‐positive partner. The majority (93%) were married and had a primary school education or lower (60%). Most were identified as PrEP candidates because of an STI or vaginal infection (96%). Thirty women completed IDIs (Table 2), of whom 6 had an HIV‐positive partner, 14 had an HIV‐negative partner and 10 did not know their partner's status.

**Table 1:** Descriptive statistics of pregnant women participating in baseline survey (*n* = 200)

	*n* (%) or Mean [SD]
Age	25.3 [5.4]
Partner HIV status (primary partner)	
*HIV‐positive*	10 (5.1%)
*HIV‐negative*	147 (74.6%)
*Unknown*	40 (20.3%)
Married	182 (92.9%)
Gestational age (weeks)	25.5 [8.8]
Parity (*n* previous live births)	1.9 [1.2]
Highest education level attained	
*No school*	9 (4.5%)
*Some primary school*	88 (43.8%)
*Completed primary school*	23 (11.4%)
*Some secondary school*	57 (28.4%)
*Completed secondary school*	21 (10.5%)
*Some tertiary school*	1 (0.5%)
*Completed tertiary school*	2 (1.0%)
Recruitment factors placing women at elevated HIV risk (past 12 months, not mutually exclusive)	
*Known HIV‐positive sex partner*	10 (5.0%)
*Sex partner with unknown HIV status*	34 (17.0%)
*Multiple sex partners*	37 (18.5%)
*STI or other vaginal infection*	192 (96.0%)

**Table 2:** Demographic description of in‐depth interview participants (pregnant PrEP users; *n* = 30)

	*n*
Reported partner HIV status	
*HIV‐positive*	6
*HIV‐negative*	14
*Unknown*	10
PrEP eligibility reasons (past 12 months, not mutually exclusive)	
*STI diagnosis*	27
*Partner of unknown HIV status*	8
*HIV‐positive partner*	7
*Multiple sexual partners*	4

All but two women interviewed had never heard of PrEP before the TP2 trial and most displayed accurate knowledge of PrEP's purpose and function (excepting points of confusion below).

### 3.2 Factors motivating PrEP use

Women surveyed reported an average of 3.7 motivations (of 7) for taking PrEP (Table 3). Common motivations included protection from HIV infection for self (100%) and baby (100%), perceived HIV risk (81%) and partner risk behaviours (73%). Similar motivations were reflected in the IDIs (below), with additional motivations related to STI diagnosis raised in IDIs that were not reflected in survey items.

**Table 3:** Pregnant women's reported PrEP motivations and concerns and their association with decisional regret in baseline survey (*n* = 200)

	*n* (%) or Mean [SD]	Decisional regret Mean (95% CI)^a^
PrEP use motivations		
(1) Wanted to protect myself from HIV infection (n=199)		
*No*	0 (0.0%)	–*
*Yes*	199 (100.0%)	
(2) Wanted to protect the baby I am expecting from HIV infection		
*No*	0 (0.0%)	–*
*Yes*	200 (100.0%)	
(3) Believed that I am at risk for HIV infection		*p=0.45*
*No*	38 (19.0%)	1.25 (1.13, 1.37)
*Yes*	162 (81.0%)	1.20 (1.15, 1.26)
(4) Concerned about my partner's risk behaviours		*p=0.93*
*No*	53 (26.6%)	1.22 (1.13, 1.31)
*Yes*	146 (73.0%)	1.21 (1.16, 1.27)
(5) Concerned that I do not know the HIV‐status of my partner(s) (n=53)		
*No*	23 (43.6%)	– ^†^
*Yes*	30 (56.6%)	
(6) Concerned that my partner's treatment (ART) will not protect me (n=10)		
*No*	3 (30.0%)	– ^†^
*Yes*	7 (70.0%)	
(7) Wanted to support my partner by taking antiretroviral drugs with him (n=10)		
*No*	5 (50.0%)	– ^†^
*Yes*	5 (50.0%)	
**Total motivations (count)** ^b^	3.7 [1.0]	B=0.00 *p=0.99*

PrEP use concerns		
(1) Concerned that PrEP could harm my body		*p=0.75*
*No*	137 (68.5%)	1.21 (1.15, 1.26)
*Yes*	63 (31.5%)	1.23 (1.14, 1.32)
(2) Concerned that PrEP could harm the baby I am expecting		*p=0.36*
*No*	128 (64.0%)	1.19 (1.14, 1.25)
*Yes*	72 (36.0%)	1.25 (1.16, 1.34)
(3) Didn't think I am at risk for HIV infection		*p=0.03*
*No*	170 (85.0%)	1.20 (1.15, 1.25)
*Yes*	30 (15.0%)	1.31 (1.18, 1.43)
(4) Concerned that my partner(s) would be upset if he/they knew I take PrEP		*p=0.03*
*No*	180 (90.0%)	1.19 (1.14, 1.24)
*Yes*	20 (10.0%)	1.41 (1.19, 1.63)
(5) Concerned that family/friends would be upset if they knew I take PrEP		*p=0.40*
*No*	178 (89.0%)	1.20 (1.15, 1.25)
*Yes*	22 (11.0%)	1.30 (1.12, 1.48)
(6) Worried people would think I was HIV‐positive if they saw me taking PrEP		*p=0.15*
*No*	153 (76.5%)	1.20 (1.14, 1.25)
*Yes*	47 (23.5%)	1.27 (1.17, 1.38)
(7) Worried people would think I was promiscuous if they knew I take PrEP		*p=0.58*
*No*	177 (88.9%)	1.20 (1.15, 1.25)
*Yes*	22 (11.0%)	1.30 (1.11, 1.49)
(8) Concerned about being able to take a pill every day		*p=0.23*
*No*	161 (80.5%)	1.19 (1.14, 1.24)
*Yes*	39 (19.5%)	1.31 (1.17, 1.44)
(9) Don't like taking pills (n=199)		*p=0.01*
*No*	173 (86.9%)	1.19 (1.14, 1.23)
*Yes*	26 (13.1%)	1.42 (1.22, 1.61)
**Total concerns (count)** ^b^	1.7 [1.9]	B=0.036 *p=0.005*

Note: Motivation 5 is applicable to 53 participants who did not know their partner's HIV status; motivations 6 and 7 apply to 10 participants who reported their partner was HIV‐positive.

^a^ *p*‐value for Mann–Whitney test (unless otherwise noted).

^b^ *p*‐value for linear regression.

* All or most participants endorsed, thus bivariate association cannot be tested.

^†^ Indicates insufficient sub‐sample to test the bivariate association.

#### 3.2.1 HIV prevention and risk perception

A general desire for HIV protection was a prominent theme in the qualitative interviews. While most women noted the importance of protecting themselves from HIV, many emphasized their desire to protect their unborn baby: *“… you protect yourself and if pregnant you protect your unborn child”* (PrEP user, partner HIV status unknown). Some women related their motivation for PrEP use to the peace of mind it would offer:

*“… you begin to really understand that without the medication we are at risk and once you are taking the medication you are more at peace knowing the PrEP is protecting you.”*

—PrEP user, partner HIV‐negative

This motivation may have been particularly strong for women in serodiscordant relationships. Many described distress upon learning their partner's HIV status and contrasted the fear of infection with the hope offered by PrEP:

*“They said that as a discordant couple, PrEP would help prevent me from acquiring HIV from my husband… That is why I chose to protect this unborn baby from HIV… I was motivated to use PrEP because there are some things that I like… not contracting HIV, not being scared, being hopeful.”*

—PrEP user, partner HIV‐positive

This relief may be connected to doubts about the protection provided by their partners’ HIV treatment; 7 of the 10 women with HIV‐positive partners surveyed reported a concern that their partners’ HIV treatment would not protect them. Though five of these women endorsed a desire to support their partner by taking ARVs with them in the survey, only one participant discussed this motivation in her interview.

A majority (57%) of the 53 survey participants who did not know their partner's HIV status were concerned about this lack of knowledge. Qualitatively, women expressed similar concerns, some signalling mistrust at their partner's avoidance of HIV testing. Women whose partners were HIV‐negative reported reassurance about their partner's test result; however, they also understood that their partners’ status could change over the course of their pregnancy and thus desired protection despite a recent negative test:

*“I thought about protecting my baby because you can test today and be negative… You can then test three months later and be found to be positive which is still before you have given birth, so I decided to take the medication to protect my baby… you can't fully trust each other, we are intimate and you cannot see when one has HIV.”*

—PrEP user, partner HIV‐negative

#### 3.2.2 STI‐related motivations

As many women were identified as PrEP candidates due to an STI diagnosis, many naturally attributed their desire for HIV protection to the risk connoted by their STI. Most displayed an understanding of the connection between STIs and HIV:

“*This was not the first time I had suffered from this STI, so I thought that since I was struggling with it and they told me that if I see that I am frequently getting this STI, I am at risk of contracting HIV*.”

—PrEP user, partner HIV‐negative

Others were confused that PrEP was part of their STI treatment: “*They said that we need to take PrEP to be protected from HIV and also to protect the unborn baby from HIV infection and candida*” (PrEP user, partner HIV‐negative). The coincidence of STI diagnosis and treatment and referral to the PrEP trial had apparently been confusing for some. Though reflecting a misunderstanding, this belief was motivating for women: *“I accepted [PrEP] because I wanted the disease [STI] to disappear and not reappear”* (PrEP user, partner HIV‐negative).

#### 3.2.3 Concerns about partner non‐monogamy

Most women interviewed mentioned known, suspected or anticipated partner non‐monogamy as a central motivation for using PrEP. Some women expressed a belief that men are expected to seek other sex partners and did not discuss specific knowledge of non‐monogamy. Others spoke in more specifics:

“*I do not know about my husband's movements and moreover he is a student and there are lots of girls at the school he is at. So, I don't know what he does, that is why I made the decision to use PrEP to protect myself*.”

—PrEP user, partner HIV‐negative

Some women discussed knowledge of current or past partner non‐monogamy, which motivated their desire for HIV protection:

“*I thought, my husband says I had girlfriend here and I had a girlfriend there. He will surely infect me with some disease someday. So now that I have this opportunity to be taking medicine, I should just thank the heavens that I will be protected from HIV*.”

—PrEP user, partner HIV status unknown

### 3.3 Concerns about PrEP use

Women's concerns when deciding to use PrEP were diverse. Women surveyed reported 1.7 concerns (of 9) on average. The most common concerns included side effects harming their baby (36%) or themselves (32%), and being perceived as HIV‐positive (24%). Similar concerns were reflected in the qualitative interviews (below).

#### 3.3.1 PrEP safety for baby

One‐third of survey participants (36%) were concerned that PrEP could harm their unborn baby. Women interviewed were primarily concerned about the risk of birth defects, miscarriage and pre‐term delivery. Often, these concerns were addressed by clinicians:

“*They said that one could give birth to a baby with defects or one with epilepsy… I felt that while I could be ending my problem I could also thereafter be struggling with the baby I would give birth to. That was the concern I took to the doctor*.”

—PrEP user, partner HIV‐positive

While this woman brought her concerns to a study physician, some rather struggled with their concerns internally or discussed their concerns with partners or friends.

#### 3.3.2 PrEP side effects and safety for self

Women interviewed discussed concerns regarding nausea, dizziness, weakness and physical appearance changes: *“Some people say that when they take this PrEP they feel dizzy, nauseous or drowsiness…”* (PrEP user, partner HIV‐negative). Others worried about more major safety concerns:

“*Taking medicine when you do not have the virus… they could damage you inside… because of the large quantity of this medicine in the body… I thought maybe [PrEP] could cause other diseases inside me [like] cancer… the liver could get damaged*.”

—PrEP user, partner HIV‐negative

This woman related her concerns about the safety of PrEP to a perceived mismatch of taking ARVs when not HIV‐positive. Others had general worries about the potential weakening effects of PrEP. Others still were concerned about changes to their appearance: *“I had some concerns… losing weight or even changing your appearance or even messing up my face… because these drugs gave me some sense like they were [ART]”* (PrEP user, partner HIV status unknown).

#### 3.3.3 Anticipated stigma and disapproval

Fears of being stigmatized as a result of PrEP use and other negative reactions from family and friends were central in many IDIs. Women worried that people would perceive their PrEP pills as antiretroviral therapy (ART). As this woman shares, she was afraid that family members or neighbours would make fun of her:

“*In the home there are many people who come and often when I am taking my medicine the people are there, and when they see, they think I have the virus while I do not. So I get concerned that people… can start mocking you that you take ARVs*.”

—PrEP user, partner HIV‐negative

Some anticipated stigmatization but asserted they were not deterred from disclosing their PrEP use. Concerns about being perceived as taking ART often co‐occurred with confusion about having been diagnosed as HIV‐positive (below). Others wanted to avoid disclosure and keep their use of PrEP secret because of anticipated disapproval or stigmatization:

“*It was difficult because I had problems and questions about where I was going to keep the drugs… the second problem was the way my family is like, what happens if they hear about this how would I stay with them?*”

—PrEP user, partner HIV‐negative

#### 3.3.4 Confusion about HIV status and the difference between PrEP and ART

Some women were confused about why they were prescribed ARVs if they were not HIV‐positive. It was apparent that they were previously familiar with ART, and the PrEP education they received had challenged their prior understanding of the purpose and use of ARVs:

“*…when they gave me [PrEP], I was afraid, I thought, ‘how do I take ARVs when I do not have the virus,’ but I just accepted… that they wanted to protect the child and me*.”

—PrEP user, partner HIV‐negative

For some, this confusion made them wonder if or believe that they had tested HIV‐positive despite the information they had received. Some overcame this misunderstanding with counselling, while others believed that they had been purposefully misled:

“*I had concerns that maybe they are not telling me the truth I have HIV and they simply don't want to tell me. Maybe these are [ART] and they worry that I am going to be very worried maybe they are hiding something*.”

—PrEP user, partner HIV‐negative

#### 3.3.5 Pill burden and attributes

Though less commonly discussed in interviews than the concerns above, several women were daunted by the prospect of taking a daily pill and were worried about their ability to adhere to PrEP. Some explained that they had never taken PrEP before, or any daily medications: *“I was like am I going to manage to take this medication because I had not taken it before”* (PrEP user, partner HIV‐positive). A few women were concerned about pill size:

“*They had mentioned that the pills were big and that I might have difficulty when swallowing them… maybe I would be feeling nausea, having trouble swallowing, but in the end I accepted it*.”

—PrEP user, partner HIV status unknown

### 3.4 Involvement and influence of others in PrEP decision‐making

While women typically indicated they had made their PrEP decisions independently, they also discussed the roles partners and family played in their decision‐making.

#### 3.4.1 Partner influence

Most women expressed that they made the decision to use PrEP on their own but had disclosed their decision to their partner (all but two women shared their decision; one was separated from her partner, and another's partner was deceased). Few women were accompanied by their partners at the clinic; most shared information about PrEP with their partners at home after enrolling. Most women who disclosed their decision to their partners did not explicitly seek partner approval, seeing the decision as theirs to make:

“*He didn't play any role, I did it all. There is a saying that ‘the one with the running stomach is the one that opens the door,’ [the one with the problem must take action] so it was not his role to play*.”

—PrEP user, partner HIV status unknown

Others sought approval from their partners for using PrEP, but only after making the initial decision themselves. Only two women reported seeking explicit permission from their partner before accepting PrEP. Most women who disclosed to their partner received supportive responses: *“When I told him, he said he had heard about this drug and it is a good drug. He said I had made a good decision”* (PrEP user, partner HIV‐negative). However, a few women received unsupportive responses: *“He was not happy when I told him… he was not open to tell me why he was not happy… he just left when I told him”* (PrEP user, partner HIV‐negative).

Even when partners were opposed to women's decisions, they did not necessarily forbid them from using PrEP: “*He did not accept this decision… he said ‘it's your decision, do whatever you feel is right’”* (PrEP user, partner HIV‐negative). Some women expressed that their partner's disapproval would not stop them from using PrEP: *“If he had refused, I would have carried on taking [PrEP] because it is one way in which I could be protected… This is my life”* (PrEP user, partner HIV‐negative).

Some women shared that their partners helped to address their concerns about PrEP. A few women had been hesitant to use PrEP but had been convinced by their partners to do so. As a result of these interactions, women discussed feeling reassured by partner encouragement and being more committed to use PrEP:

“*I felt that even though I had made the decision to take the medicine it was not wholehearted until my husband saw the medicine, read about it and read the form to see what it was for. That was when I felt good about taking the medicine*.”

—PrEP user, partner HIV‐negative

#### 3.4.2 Family influence

Very few women discussed PrEP with other family members before making their decision. Some were worried about how their family would react because they believed they would also have to disclose their STI diagnosis: *“They [my family] do not know… If everyone knew, they would be saying that we [my husband and I] have a certain disease [STI]”* (PrEP user, partner HIV‐negative).

Others did not involve their family because they worried about misunderstandings, especially about their HIV status: *“No one [in my family] knows… they stay far away and might think that I have HIV”* (PrEP user, partner HIV status unknown). Only a couple of women involved family members in the decision. One woman's parents were already informed of her STI diagnosis, so it was natural to tell them about PrEP:

“*I went to discuss [PrEP] with… my parents, because my parents knew about my illness [STI]. They told me it's okay to receive the drugs but I should not forget to take them*.”

—PrEP user, partner HIV‐negative

### 3.5 Feelings about PrEP decision

Despite women's diverse concerns for PrEP use, most women described the decision to use PrEP as easy, because they were excited about the HIV prevention benefits. For women who held concerns about using PrEP, most explained that their desire for protection against HIV outweighed their concerns: *“… if I am to protect myself and the baby, it is better that I use this PrEP”* (PrEP user, partner HIV‐negative). Some women expressed that they wanted to take advantage of the opportunity to use PrEP in the trial. Others added that study staff explained things well and they had the information needed to make an appropriate decision:

“*It took me time to understand, but with the way the people were talking to me and explaining things, it was just fine, there was no doubt*.”

—PrEP user, partner HIV‐negative

A few women shared they had experienced indecision or difficulty in their decision‐making because PrEP was new to them. Yet, they found it hard to say no to the HIV protection offered by PrEP:

“*I was thinking, could this medicine really help? Then later on I realized that if I ignored this medicine it would not do any good to my health, which is why I decided to make the decision to take the medicine*.”

—PrEP user, partner HIV status unknown

#### 3.5.1 Decisional regret

**Figure 1:**
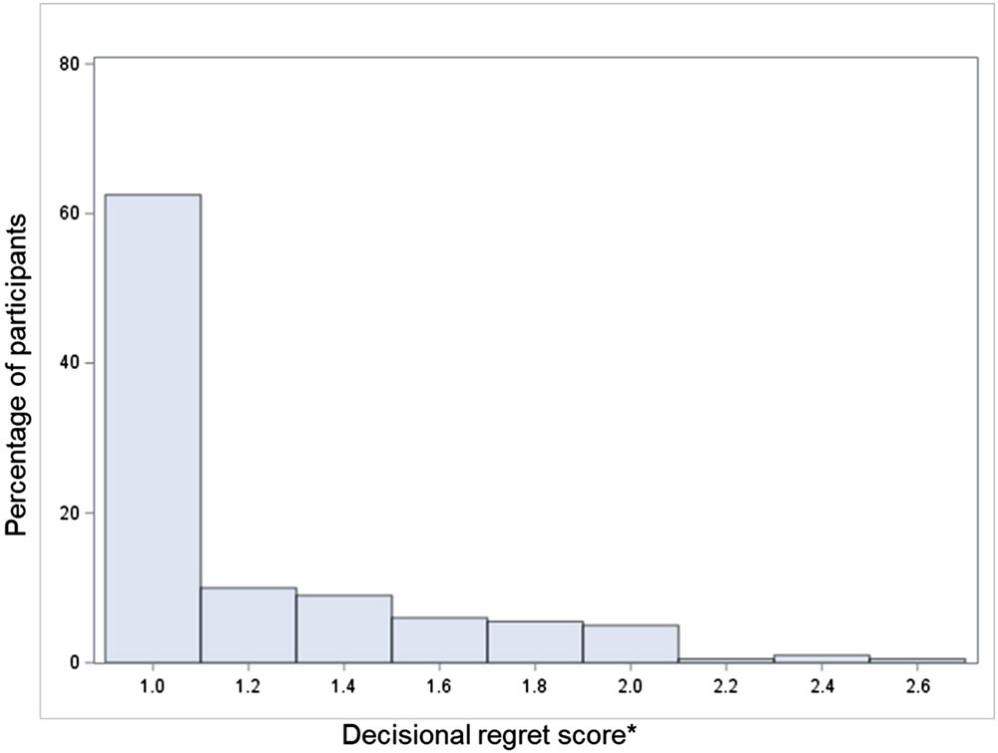
Distribution of Decisional Regret Scores (*n* = 200). ^*^Theoretical range 1–5; greater scores indicate greater regret about the decision to use PrEP.

While some women qualitatively reported struggling with the decision, in the survey, overall regret about the decision was low. Women surveyed had a mean score of 1.2 on the Decisional Regret Scale (theoretical range: 1–5, higher scores indicate greater regret). One hundred and twenty‐five women (63%; Figure 1) had the lowest possible score (1.0) and 71 scored between 1.0 and 2.0 (36%; reflecting moderately positive feelings about the decision). Only four participants scored higher than 2.0 (2.2–2.6).

Though reported decisional regret was low, endorsing a greater number of concerns was associated with greater decisional regret (Table 3: B = 0.036; *p* = 0.005). Additionally, three specific concerns were associated with greater decisional regret. Thirty women (15%) reported no perceived HIV risk and reported greater decisional regret (mean of 1.3 vs. 1.2; *p* = 0.03). The 20 women (10%) who were concerned that their partner would be upset about their PrEP use also reported greater decisional regret (mean of 1.4 vs. 1.2; *p* = 0.03). Finally, dislike of taking pills was associated with greater decisional regret (mean of 1.4 vs. 1.2; *p* = 0.01) and was reported by 26 women (13%).

## 4 DISCUSSION

Pregnant women in our study were motivated to use PrEP for HIV prevention yet harboured concerns about PrEP. Below, we discuss the implications of our results to inform PrEP decision counselling that centres women's decision‐making needs and values.

Women's perceived need for HIV protection was often motivated by an STI or concerns about partner non‐monogamy. Motivations regarding partner non‐monogamy may be related to expectations of women in this context to abstain from sex leading up to delivery and/or through 6 months postpartum, a time during which men might be expected to seek other sex partners [28, 29]. Though women mostly understood PrEP's function, some incorrectly believed that PrEP could treat or prevent STIs and described this as motivating their PrEP use. When PrEP referral follows an STI diagnosis, initial and repeated clarification of the distinction between STI treatments and PrEP is critical to avoid confusion.

Women's primary concerns were related to side effects causing adverse birth outcomes or harming wellbeing. Side effect‐related concerns are noted in other studies as a possible deterrent for women [30, 31]. Some side effects that concerned women are not known to be associated with TDF/FTC but rather other antiretroviral medications, including lipodystrophy and liver toxicity. Some women may also conflate PrEP side effects with pregnancy symptoms [32]. Regardless of attribution, perceived side effects may be linked to early discontinuation [33, 34, 35], or non‐adherence [36, 37]. Because common side effects typically resolve within weeks, early and frequent opportunities for women to voice concerns, reminders that side effects should resolve and information about which commonly feared side effects do not occur with PrEP are important.

Anticipated stigmatization was also an important concern, with women worrying that people would mistake their PrEP for ART and infer that they are HIV‐positive. For some, this anticipated stigma was related to their own confusion about the difference between PrEP and ART. In contexts like Malawi where the adult HIV prevalence approaches 9% [38], familiarity with ART is likely high, while familiarity with novel interventions like PrEP is likely low [39, 40, 41]. While communicating that TDF/FTC has been used safely as ART may provide safety assurances, it may also lead to confusion about women's true HIV status and raise anticipated stigma [42, 43, 44]. Comparisons between PrEP and ART regimens should be included in patient education with caution. Stigma‐related concerns may discourage PrEP uptake, but concealment strategies and facilitated disclosure may mitigate anticipated stigma [42, 45, 46].

Some women were also concerned about partner reactions to their PrEP use. Direct partner education may help address these concerns [47], and relieve women of the burden of sharing information second‐hand. Though most women had independently decided to use PrEP, there was diversity in partner involvement both before and after the initial decision. Promoting male partner co‐education and—if the woman desires—involvement in decision‐making may strengthen partner support for PrEP use and facilitate adherence [48, 49, 50].

Most women did not involve or share their decision with family members, and some were concerned about having to disclose their STI diagnosis when explaining their PrEP use. We have not seen this concern in previous studies, and this finding is likely related to the fact that most women were eligible because of an STI. This concern may arise elsewhere if PrEP is offered in the context of an STI diagnosis. In another departure from prior studies, women in other settings have expressed concerns about perceived promiscuity if known to use PrEP. While we asked women about this in the survey, none of the women interviewed raised concerns related to perceived promiscuity.

Despite concerns, women generally felt positively about their decision to use PrEP and reported low decisional regret. Women with reservations found that the HIV protection PrEP offered outweighed their concerns. Still, women with more concerns had more decisional regret, and concerns related to partner disclosure, dislike of pills and no perceived HIV risk were associated with decisional regret. Though assessed shortly after women made their decision, our decisional regret findings provide evidence of early feelings about the perceived appropriateness of PrEP use, which merit future longitudinal investigation. These findings can be used in combination with qualitative evidence to understand the potential salience of different values in women's decision‐making to inform decision counselling materials for future testing.

The fact that some women harboured concerns about PrEP even after initial counselling suggests the need for better patient engagement in initial decision‐making and counselling about PrEP to ensure user fit and comfort through approaches like SDM [21]. By providing patients with knowledge about the options available to them, and helping them to clarify their needs and values, SDM may reduce regret or uncertainty around the decision to start PrEP [51], and improve adherence by ensuring user fit and motivation [52, 53, 54, 55, 56]. The information on women's PrEP decision‐making values will be used to inform an SDM approach for women considering PrEP use during pregnancy.

### 4.1 Limitations

The results of this study should be interpreted with key limitations in mind. Participants’ reports of PrEP motivations, concerns and regret about starting PrEP were likely susceptible to social desirability bias as women were participating in a PrEP adherence study. Women purposively recruited for IDIs may not be representative of those who chose not to participate. Participants were recruited from urban and peri‐urban settings in Lilongwe, thus findings are primarily generalizable to women in similar settings in the region. All women included in this study had accepted PrEP, thus their perspectives cannot represent those of women who decline PrEP. Data were collected after women had accepted PrEP, with quantitative assessments of concerns and decisional regret occurring at the same visit after women made the decision to start PrEP and before they initiated PrEP. Future longitudinal studies are needed to better understand the relationship between concerns and decisional regret about PrEP use over time, with repeated assessments of decisional regret after PrEP initiation. A strength of the present study compared to those previous is that the discussion of PrEP decision‐making was real rather than hypothetical.

## 5 CONCLUSIONS

Pregnant women initiating PrEP were motivated by the promise of HIV protection for themselves and their baby which they often desired because of the risk connoted by an STI or concerns about partner non‐monogamy. This motivated them to accept PrEP despite concerns about safety and side effects, stigmatization, adherence burden and pill attributes. Most women felt positively about the decision to start PrEP, but those with more concerns and those concerned about partner disclosure, disliking pills or with no perceived HIV risk reported higher levels of regret about the decision. Centring women's preferences and concerns through an SDM approach may help to identify and address initial concerns about PrEP use and support prevention‐effective PrEP use in pregnant women.

## COMPETING INTERESTS

LMH and CEG report grant support from Gilead Sciences. This does not alter our adherence to journal policies on sharing data and materials. The authors otherwise have declared that no competing interests exist.

## AUTHORS’ CONTRIBUTIONS

LMH conceptualized and designed the study, led the analysis and wrote the manuscript. CEG contributed to study conceptualization and design and mentored LMH. FS contributed to the study design and oversaw the study conducted at the research site. TP coordinated the study and contributed to qualitative analysis. JT collected qualitative data and contributed to qualitative analysis. AY contributed to qualitative analysis. LDP contributed to study design and mentored LMH. SM contributed to study design and mentored LMH. BHC contributed to study conceptualization and design, mentored LMH and co‐led the parent study with WM. WM contributed to study conceptualization, mentored LMH and co‐led the parent study with BHC. All authors contributed to the writing of the manuscript and approved the final manuscript for publication.

## FUNDING

This study was funded by the National Institute of Mental Health (K01 MH121186) and the National Institute of Allergy and Infectious Diseases (R01 AI131060). Additional investigator and administrative support is provided by NIAID (K24 AI120796, P30 AI050410) and Fogarty International Center (D43 TW009340, D43 TW010060). Funders were not involved in the study design development, writing of the protocol and in the decision to submit this article for publication.

## Data Availability

The data that support the findings of this study may be available on request. The data are not publicly available due to privacy or ethical restrictions.
